# Feasibility and safety of an analgesia-first strategy without hypnotic sedatives in adult patients admitted to the intensive care unit after neurosurgical craniotomy: a protocol for a single-arm, single-center exploratory prospective study

**DOI:** 10.3389/fmed.2026.1848074

**Published:** 2026-06-10

**Authors:** Yu-Mei Wang, Ying Tian, Shu-Ya Wang, Guang-Qiang Chen, Guang-Zhi Shi

**Affiliations:** Department of Critical Care Medicine, Beijing Tiantan Hospital, Capital Medical University, Beijing, China

**Keywords:** analgesia and sedation, analgesia-first, neurosurgical craniotomy, remifentanil, study protocol

## Abstract

**Introduction:**

Postoperative neurosurgical patients often require pain management and agitation control while still being assessed for consciousness and focal neurological signs. General ICU trials suggest that minimizing the use of hypnotic sedatives may be feasible for selected mechanically ventilated patients; however, neurocritical care patients may have different safety requirements. This study aims to explore the feasibility and safety of an analgesia-first strategy, without the routine use of hypnotic sedatives, in adult patients admitted to the ICU after neurosurgical craniotomy.

**Methods and analysis:**

This is a single-center, single-arm, open-label, exploratory prospective study. A total of 65 adult patients after neurosurgical craniotomy with an anticipated ICU stay of more than 24 h and Richmond Agitation-Sedation Scale (RASS) score ≥ + 1 will be enrolled, provided that they do not require deep sedation. Eligible patients will receive protocolized nonpharmacological measures and remifentanil-based analgesia titrated from 0.1 to 0.2 μg/kg/min every 10–15 min to achieve a target RASS score of −2 to +1 and Critical-Care Pain Observation Tool (CPOT) score of 0–1. Rescue sedation with midazolam or propofol is permitted when clinically required for safety. The primary endpoint is successful protocol management during the first 24 h after initiation, defined as absence of protocol failure caused by rescue hypnotic sedative use, sustained RASS scores outside the target range, a serious safety event requiring protocol discontinuation, or clinical deterioration. Key safety endpoints include significant agitation, accidental extubation, accidental catheter or drain removal, respiratory depression, severe hypotension or bradycardia, delirium, opioid-induced rigidity, neurological deterioration, seizures, emergent neurosurgical intervention, and urgent unplanned computed tomography (CT) or magnetic resonance imaging (MRI) for suspected neurological worsening. Secondary endpoints include analgesic and sedative exposure, nursing workload, bispectral index (BIS) values, duration of intubation, ventilator-free days through day 28, ICU and hospital length of stay, ICU costs, pneumonia, and in-hospital mortality.

**Clinical trial registration:**

ClinicalTrials.gov, identifier NCT06727435.

## 1 Introduction

Analgesia and sedation are core components of intensive care unit (ICU) care. They are used to relieve pain and anxiety, prevent harmful physiological stress responses, improve tolerance of invasive devices and mechanical ventilation, and reduce noxious stimulation (1–3). Contemporary ICU practice has moved away from deep continuous sedation to analgesia-first care, lighter sedation targets, delirium prevention, early mobilization, and patient-centered comfort strategies (2–5).

Patients admitted to the ICU after neurosurgical craniotomy present a specific challenge. Postoperative agitation after intracranial surgery may lead to unplanned extubation, catheter or drain removal, injury, hypertension, coughing, increased sympathetic activation, and potentially adverse neurological consequences (6, 7). The incidence of agitation after elective intracranial operations was 29%, which is higher than that previously observed in other surgical populations (6). Neurosurgical patients may be more vulnerable to stress caused by agitation due to factors such as longer anesthesia duration, delayed extubation, pain, and post-craniotomy frontal pneumocephalus (7, 8). Brain lesions and intracranial manipulations in neurosurgical patients might affect brain regions involved in cognition and emotion and are assumed to influence postoperative cognition function (6). At the same time, excessive hypnotic sedation may obscure the level of consciousness, pupillary and focal neurological examinations, early seizures, intracranial hypertension, or surgical complications (9–13). Previous neurosurgical studies have reported that postoperative agitation and delirium after craniotomy are not uncommon, and risk factors, including longer anesthesia duration, delayed extubation, pain, and postoperative pneumocephalus, have been identified (6–8).

However, investigations regarding analgesia and sedation in neurosurgical populations have been inadequate (2–4, 14–17). Neurosurgical patients have often been excluded from studies of analgesia and sedation in general ICU populations (2–4, 14). This population requires frequent assessments of consciousness and neurological signs, making analgesia and sedation management difficult and complex. Several general ICU studies support interest in minimizing routine hypnotic sedation. In a single-center randomized trial, a protocol of no sedation with morphine boluses increased ventilator-free days and shortened ICU and hospital stays compared to sedation and daily interruption, although agitated delirium was more frequent (14). In the larger multicenter NONSEDA trial (15), mortality at 90 days did not differ significantly between non-sedation and light sedation with daily interruption, and many patients in the non-sedation group still required sedatives during ICU stay, most commonly because of delirium. Related NONSEDA substudies have also emphasized that the effects of non-sedation on physical function and cognition require dedicated evaluation (16, 17).

These findings cannot be directly transferred to post-craniotomy neurocritical care. Reviews, consensus statements, and observational studies in neurocritical care have highlighted that patients with brain injury have distinct indications for sedation, including intracranial pressure regulation, seizure control, reduction of cerebral oxygen consumption, ventilator synchrony, and management of severe agitation (9–13). Observational data from brain–injury ICUs demonstrate wide variation in sedative and analgesic practices and support the structured use of sedation and pain scales (11). Expert consensus also supports administering analgesics before sedatives when clinically appropriate but acknowledges the limited high-quality evidence available in this population (10, 13). Therefore, an analgesia-first strategy without routine hypnotic sedatives should be evaluated cautiously with explicit neurological safety monitoring and predefined rescue criteria.

Remifentanil is a short-acting opioid with a rapid onset and offset because it is metabolized by non-specific blood and tissue esterases (18). These pharmacological properties make it suitable for titrated analgesia when repeated neurological assessments are required (18–21). We therefore designed this single-arm exploratory study to estimate the feasibility and safety of a remifentanil-based analgesia-first strategy without routine hypnotic sedatives in selected adult patients admitted to the ICU after neurosurgical craniotomy.

## 2 Materials and methods

### 2.1 Study design and trial status

**Figure 1:**
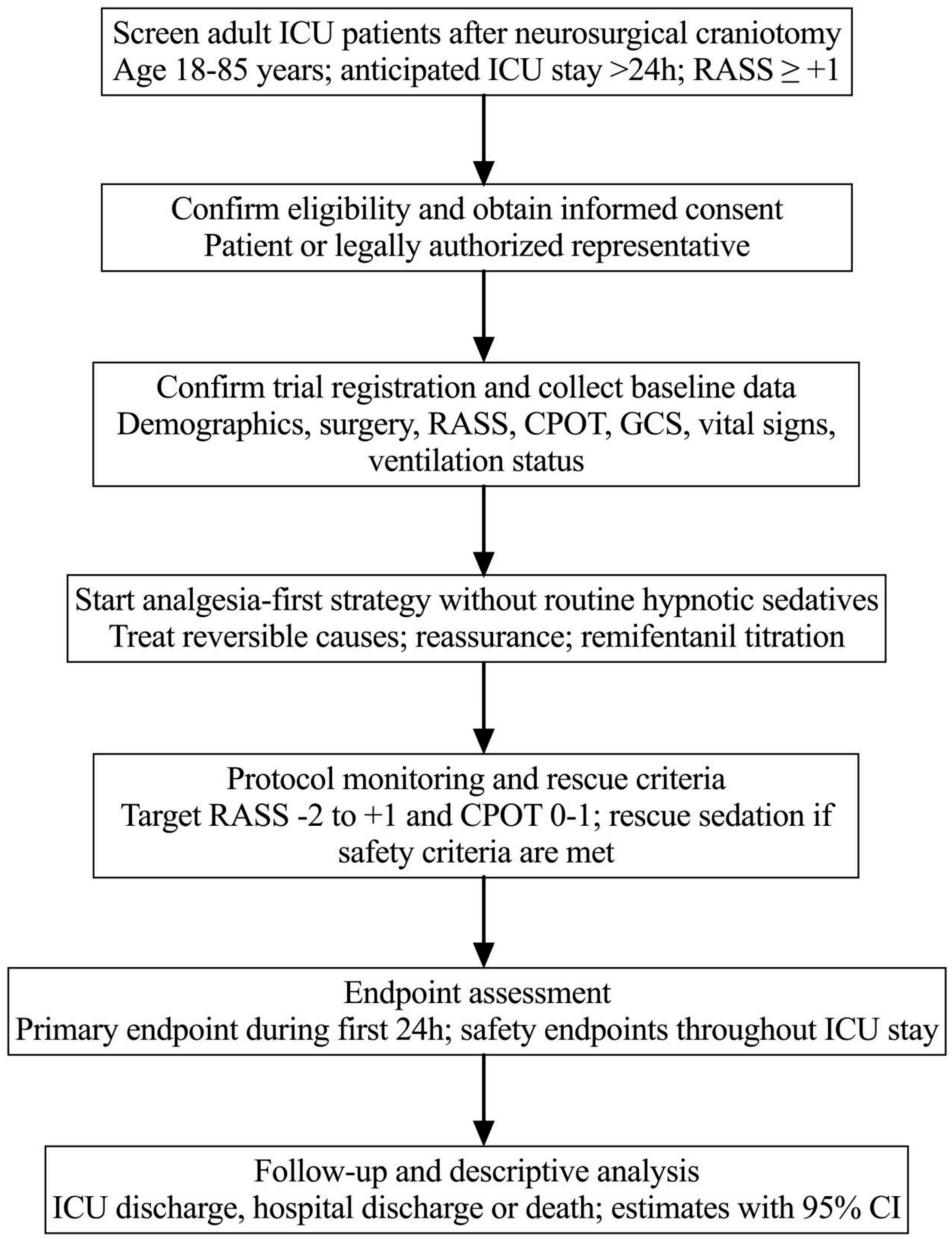
Flowchart of the study design. ICU, intensive care unit; RASS, Richmond Agitation-Sedation Scale; CPOT, Critical-Care Pain Observation Tool; GCS, Glasgow Coma Scale.

This is a single-center, single-arm, open-label, exploratory prospective clinical study conducted in the Department of Critical Care Medicine, Beijing Tiantan Hospital, Capital Medical University, China, which is a major neurosurgical center. The ICU mainly receives patients after neurosurgical procedures. The study is reported according to the Standard Protocol Items: Recommendations for Interventional Trials (SPIRIT) reporting guidelines. A SPIRIT checklist is submitted as Supplementary material. The flow chart is shown in Figure 1, and the assessment schedule is shown in Table 1.

**Table 1:** Schedule of enrollment, intervention, assessments, and follow-up.

Assessment	Screening/Enrollment	0–4 h after initiation	Every 8 h during protocol	Daily in ICU	ICU discharge	Hospital discharge/Death
Eligibility and consent	X					
RASS and CPOT	X	15, 30, 60, 120, 240 min	X	X	X	
Vital signs and respiratory monitoring	X	X	X	X	X	
GCS and neurological examination	X	X	X	X	X	
BIS	X	X	X	X		
CAM-ICU				07:00 and 21:00	X	
Drug exposure and dose adjustments		X	X	X	X	
Adverse events and neurological safety events		X	X	X	X	
ICU outcomes and cost					X	
Hospital outcomes and mortality						X

ICU, intensive care unit; RASS, Richmond Agitation-Sedation Scale; CPOT, Critical-Care Pain Observation Tool; GCS, Glasgow Coma Scale; BIS, bispectral index; CAM-ICU, Confusion Assessment Method for the Intensive Care Unit.

The study is registered at ClinicalTrials.gov (NCT06727435). The registry currently lists the study as “not yet recruiting,” with the original estimated dates and original title. Recruitment is scheduled to start in August 2026 after the publication of this protocol. An updated ClinicalTrials.gov record was submitted on 20 May 2026 to align the registry with the revised title, exploratory study design, primary endpoint, recruitment status, sample size, and timeline. Public posting of the updated registry record may require registry processing. The registry entry will be updated before enrollment and publication to align with the revised title, exploratory study design, primary endpoint, recruitment status, and timeline.

### 2.2 Informed consent

All patients admitted to the ICU after neurosurgical craniotomy will be screened. Written informed consent will be obtained preoperatively whenever feasible for patients undergoing planned craniotomy. For patients who lack decision-making capacity postoperatively, consent may be obtained from a legally authorized representative according to IRB-approved procedures and local regulations. Capacity will be assessed by the treating clinician and research team before consent. Patients who regain capacity after representative consent will be informed about the study and asked to confirm continued participation. Deferred consent will not be used unless specifically permitted by the IRB. Patients or legally authorized representatives may withdraw from the study at any time without affecting clinical care.

### 2.3 Inclusion criteria

1. Age 18–85 years;
2. Admission to the ICU after neurosurgical craniotomy;
3. Anticipated ICU stay of more than 24 h according to the treating team at ICU admission or screening;
4. The Richmond Agitation-Sedation Scale (RASS) score ≥ + 1, indicating restlessness or agitation requiring clinical management;
5. RASS score, Critical-Care Pain Observation Tool (CPOT) score, and neurological status can be assessed reliably for protocol monitoring.

### 2.4 Exclusion criteria

1. Need for deep sedation or therapeutic coma, including partial pressure of arterial oxygen/fraction of inspired oxygen (PaO_2_/FiO_2_) ≤ 100 mmHg, neuromuscular blockade requiring unconsciousness, status epilepticus, mandatory immobility for surgical or procedural safety, severe traumatic brain injury with intracranial hypertension, therapeutic hypothermia, or any clinical condition requiring a RASS score < −2 (12, 13);
2. Medullary lesion, brainstem condition, or other disorder associated with impaired respiratory drive in which opioid analgesia is judged unsafe by the treating physician (22);
3. Inability to assess the RASS score or neurological status due to coma, severe aphasia, status epilepticus, severe cognitive dysfunction, schizophrenia, mania, or another psychiatric or neurological condition that precludes reliable assessment;
4. Use of sedatives or opioid analgesics ≥ 1 week before enrollment;
5. Expected ICU stay time of ≤ 24 h;
6. Delirium, alcohol withdrawal, active severe psychiatric illness, or ongoing antipsychotic therapy before enrollment;
7. Severe hepatic dysfunction (Child-Pugh class C);
8. Renal failure requiring renal replacement therapy;
9. Need for major surgery during the ICU stay, except short bedside or minor procedures, such as lumbar puncture or ventricular drainage;
10. Known allergy or contraindication to remifentanil, morphine, midazolam, propofol, or other protocol medications;
11. Pregnancy or lactation;
12. Participation in another interventional clinical trial that could interfere with the study intervention or outcomes;
13. Patient or legally authorized representative unwilling to participate;
14. Investigator judgment that the inclusion is inappropriate because of a specific safety concern documented in the screening record.

### 2.5 Intervention

The study intervention is an analgesia-first strategy without routine hypnotic sedatives. The strategy does not imply the absence of all sedative effects; remifentanil may influence arousal, and the RASS target includes light sedation (19, 20, 23). Routine hypnotic sedatives are avoided unless rescue sedation is clinically required.

RASS and CPOT scores will be assessed routinely, with target RASS -2 to +1 and CPOT 0–1 (23, 24). The intervention sequence is summarized in Table 2. Reversible causes of discomfort will first be evaluated and treated, including hypoxia, airway obstruction, ventilator dyssynchrony, pain, urinary retention, nausea, tube irritation, environmental stressors, and neurological deterioration. Non-pharmacological measures such as reassurance, communication, family support when appropriate, sleep protection, and mobilization when safe will be used before pharmacological escalation (2–4, 10).

**Table 2:** Intervention algorithm for the analgesia-first strategy without routine hypnotic sedatives.

Step	Action	Protocol rule
1	Assess and treat reversible causes of discomfort, agitation, pain, hypoxia, tube obstruction, urinary retention, nausea, environmental stressors, and neurological change.	Treat the cause first; obtain urgent neurological evaluation if deterioration is suspected.
2	Use non-pharmacological reassurance, communication, sleep protection, family support when feasible, and mobilization when safe.	Proceed to remifentanil if the RASS score remains ≥ + 1 and medication is required.
3	Start remifentanil at 0.10–0.15 μg/kg/min.	Monitor the RASS score, CPOT score, respiratory status, hemodynamics, and neurological status continuously or at scheduled time points.
4	Titrate remifentanil every 10–15 min by 0.025 μg/kg/min to a maximum of 0.20 μg/kg/min.	Target RASS -2 to +1 and CPOT 0–1; reduce, interrupt, or discontinue for safety events.
5	Administer intravenous morphine 2.5 mg or 5 mg for breakthrough pain when clinically indicated.	Repeated rescue is defined as ≥ 3 boluses/6 h or ≥ 4 boluses/24 h; persistent CPOT > 1 despite rescue is failure.
6	Use rescue midazolam or propofol if safety criteria are met.	Classify as non-success for the primary endpoint and document the indication.

CAM-ICU, Confusion Assessment Method for the Intensive Care Unit; CPOT, Critical-Care Pain Observation Tool; ICU, intensive care unit; RASS, Richmond Agitation-Sedation Scale.

If the RASS score remains ≥ + 1 and pharmacological treatment is required, remifentanil will be initiated at 0.10–0.15 μg/kg/min. The dose will be titrated every 10–15 min by 0.025 μg/kg/min to a maximum of 0.20 μg/kg/min to achieve a target RASS score of −2 to +1 and CPOT score of 0–1 (19, 20, 23, 24). Continuous peripheral oxygen saturation (SpO_2_), respiratory rate, heart rate, and non-invasive or invasive blood pressure monitoring will be required during continuous remifentanil infusion. For non-ventilated patients, continuous pulse oximetry and respiratory rate monitoring are mandatory; capnography will be used when available or clinically indicated. For mechanically ventilated patients, ventilator synchrony and apnea alarms will also be monitored.

Intravenous morphine bolus doses of 2.5 mg or 5 mg may be administered for breakthrough pain when the CPOT remains > 1 or when the treating clinician judges that pain is contributing to agitation despite remifentanil titration (9). Repeated morphine rescue is defined as ≥ 3 boluses within 6 h or ≥ 4 boluses within 24 h. The repeated use of morphine for rescue, along with a persistent CPOT score >1, persistent RASS scores outside the target, or the need for hypnotic rescue sedation will be classified as protocol failure for the primary endpoint. If the persistent CPOT score remains > 1 despite maximum remifentanil and morphine rescue, it will also be classified as protocol failure, even if hypnotic sedation is not used.

Rescue hypnotic sedation using midazolam or propofol is permitted when necessary for safety and clinical reasons. The criteria for its use include persistent RASS scores of +3 to +4 despite maximum protocolized analgesia, risk of self-injury or removal of medical devices, severe ventilator dyssynchrony, inability to maintain oxygenation or airway safety, seizures, suspected intracranial hypertension, acute neurological deterioration, requirement for urgent imaging or procedures, prone positioning, or the treating clinician’s judgment that deeper sedation is required. Any use of rescue hypnotic sedation will be recorded and counted as non-success for the primary endpoint.

The primary endpoint window is the first 24 h after protocol initiation. If the patient remains in the ICU after 24 h and continues to require analgesia, remifentanil may be continued according to the same safety protocol while clinically indicated. Daily review will determine whether remifentanil can be tapered or discontinued. Remifentanil will be stopped when analgesia is no longer required, protocol failure happens, a safety indication for discontinuation emerges, ICU discharge occurs, or the treating team determines that conventional analgesia and sedation care is required.

Delirium will be assessed using the Confusion Assessment Method for the Intensive Care Unit (CAM-ICU) at 07:00 and 21:00 daily (25). Initially, non-pharmacological methods will be employed for managing delirium. If pharmacological treatment is required, haloperidol or olanzapine may be administered according to local practice. The use of antipsychotics for delirium will be recorded as concomitant pharmacological treatment and will not be counted as hypnotic rescue sedation for the primary endpoint, but it will be considered in sensitivity analyses.

### 2.6 Outcomes

The primary endpoint is successful protocol management during the first 24 h after the initiation of the analgesia-first strategy. A successful protocol management requires no rescue hypnotic sedative use, no protocol discontinuation for safety reasons, and adequate RASS control based on the following operational rules: at least 80% of scheduled RASS assessments during the first 24 h must be within the target range of −2 to +1, with no sustained RASS > + 1 lasting more than 30 min despite protocolized treatment and no RASS score of +3 or +4 at any scheduled or clinically indicated assessment. A single brief RASS +2 episode that resolves within 30 min without rescue hypnotic sedation will not automatically count as failure but will be recorded as transient out-of-target agitation. Patients will be classified as non-success if they: receive midazolam, propofol, or other hypnotic sedatives, discontinue remifentanil because of a safety event, require escalation to deep sedation, withdraw from the protocol before endpoint ascertainment, die, or cannot complete the endpoint window due to clinical deterioration. If a patient is discharged from the ICU before 24 h without rescue hypnotic sedative use and all available RASS assessments falling within the target range, the case will be counted as success in the main analysis and evaluated in sensitivity analyses.

Key safety endpoints include accidental extubation, accidental catheter or drain removal, significant agitation, delirium, severe hypotension, severe bradycardia, respiratory depression, vomiting or aspiration risk, opioid-induced rigidity, seizure, neurological deterioration, suspected or confirmed intracranial hypertension, emergent neurosurgical intervention, and urgent unplanned CT or MRI performed for suspected neurological worsening. Urgent CT or MRI is treated as a trigger or consequence of suspected neurological deterioration rather than an adverse event itself.

Secondary endpoints include total remifentanil dose, morphine bolus dose and frequency, rescue sedative dose and frequency, nursing workload measured by a number of analgesic or sedative dose adjustments, bispectral index (BIS) values, duration of intubation, ventilator-free days through day 28, ICU length of stay, hospital length of stay, ICU cost, pneumonia, and in-hospital mortality. Duration of intubation will be calculated only for patients intubated at enrollment or intubated after enrollment; non-intubated patients at enrollment who are never intubated will be reported separately and will not be assigned an intubation duration of zero. Ventilator-free days will be defined as days alive and free from invasive mechanical ventilation from enrollment through day 28; patients who die before day 28 will be assigned zero ventilator-free days. Pneumonia will be diagnosed according to the treating team’s clinical diagnosis supported by radiographic evidence and microbiological or laboratory findings when available. ICU cost will be extracted from the hospital billing system in Chinese yuan from the hospital-care perspective. Nursing workload will be measured by the number of protocol-related analgesic or sedative dose adjustments, rescue medication administrations, and additional bedside safety interventions recorded in the case report form. The endpoint hierarchy is shown in Table 3.

**Table 3:** Endpoint hierarchy and definitions.

Category	Endpoint	Definition or assessment window
Primary feasibility endpoint	Successful protocol management	First 24 h: no rescue hypnotic sedative, no safety-related discontinuation, ≥ 80% scheduled RASS assessments within −2 to +1, no sustained RASS > + 1 > 30 min despite treatment, and no RASS score of +3 or +4.
Key safety endpoint	Significant agitation	RASS > + 2, RASS > + 1 lasting > 30 min despite treatment, or agitation requiring urgent intervention to protect the patient, airway, or devices.
Key safety endpoint	Accidental extubation	Unplanned removal of an endotracheal tube or tracheostomy tube. Reintubation and timing will be recorded.
Key safety endpoint	Accidental catheter/drain removal	Unplanned removal of CVC, arterial catheter, gastric tube, urinary catheter, surgical drain, ventricular drain, or other clinically relevant device. Replacement and timing will be recorded.
Neurological safety endpoint	Neurological deterioration	GCS decrease ≥ 2 points, new focal deficit, seizure, suspected intracranial hypertension, clinically significant new CT/MRI finding, or emergent neurosurgical intervention.
Respiratory/hemodynamic safety endpoint	Respiratory depression, severe hypotension, severe bradycardia	Protocol-defined events requiring dose reduction, interruption, discontinuation, or clinical rescue treatment.
Secondary endpoint	Clinical course and resource use	Drug exposure, dose adjustments, BIS values, intubation duration, ventilator-free days through day 28, ICU/hospital length of stay, ICU cost, pneumonia, nursing workload, and mortality.

BIS, bispectral index; CPOT, Critical-Care Pain Observation Tool; CT, computed tomography; CVC, central venous catheter; GCS, Glasgow Coma Scale; ICU, intensive care unit; MRI, magnetic resonance imaging; RASS, Richmond Agitation-Sedation Scale.

### 2.7 Data collection and assessment schedule

Data will be collected prospectively by trained research staff using standardized case report forms. Baseline data include age, sex, body mass index, smoking and alcohol history, primary diagnosis, comorbidities, preoperative neurological status, surgery type, surgical site and approach, operation duration, indwelling tubes or drains, postoperative CT findings including pneumocephalus, ventilation status, and baseline RASS, CPOT, GCS, vital signs, and blood gas analysis (BGA) results, as clinically indicated.

After the initiation of remifentanil, heart rate (HR), respiratory rate (RR), systolic blood pressure (SBP), diastolic blood pressure (DBP), mean arterial pressure (MAP), SpO_2_, RASS score, CPOT score, the Glasgow Coma Scale (GCS), BIS value (if available), respiratory status, ventilator synchrony if applicable, BGA (as clinically indicated), remifentanil dose, morphine bolus use, rescue sedative use, dose adjustments, and adverse events will be recorded at 15 min, 30 min, 1 h, 2 h, 4 h, and then every 8 h during protocol treatment. Once the target RASS score has been achieved and remains stable, routine data will be recorded at least every 24 h until ICU discharge, discontinuation of analgesia/sedation, protocol failure, withdrawal, death, or the need for deep analgesia and sedation. CAM-ICU will be used twice daily. Patients will be followed until hospital discharge or death, whichever occurs first.

### 2.8 Adverse events, rescue management, and safety oversight

Adverse events potentially associated with remifentanil or the study protocol included delirium, severe hypotension, severe bradycardia, respiratory depression, constipation, shivering, vomiting, opioid-induced muscle rigidity, accidental extubation, accidental catheter or drain removal, aspiration, and neurological deterioration. Adverse events will be graded based on severity, seriousness, expectedness, and relatedness to the intervention. The Clavien–Dindo classification will not be used as the primary adverse-event grading system for drug-related or ICU sedation-related events, although surgical complications may be described separately when relevant.

1. Delirium will be diagnosed using CAM-ICU. Initial management will emphasize non-pharmacological measures; antipsychotic medication may be used according to local clinical practice when required for patient safety. It is recommended to start with olanzapine 1.25–2.5 mg once daily, followed by dose adjustment to 1.25–20 mg once daily according to symptoms (25).
2. Severe hypotension is defined as SBP < 80 mmHg, an SBP decrease of > 20% from preinfusion baseline, or DBP < 50 mmHg. Management includes reduction or discontinuation of analgesic drugs, fluid therapy as clinically indicated, and norepinephrine infusion if needed.
3. Severe bradycardia is defined as HR < 40 beats/min or a decrease of > 20% from baseline. Management includes remifentanil dose reduction or interruption and administration of atropine or other treatment if required.
4. Respiratory depression is defined as RR < 10 breaths/min, recurrent apnea alarms, clinically significant hypoventilation, hypoxemia, hypercapnia, or the need for airway or ventilatory intervention. Management includes remifentanil dose reduction or interruption, airway support, ventilator adjustment, and BGA assessment when clinically indicated.
5. Constipation is defined as fewer than three bowel movements per week or a difficult bowel movement requiring treatment. Management includes standard bowel-regimen measures, stool softeners, laxatives, suppositories, or enemas when clinically indicated.
6. Shivering is defined as rapid rhythmic (9–12 contractions/min) skeletal muscle activity. Treatment measures include warming, warming of intravenous fluids when clinically indicated, and intramuscular promethazine 25 mg.
7. Vomiting will be recorded when clinically observed. Management includes reducing or interrupting remifentanil when drug-related vomiting is suspected, aspiration precautions, and intravenous metoclopramide 10 mg or other antiemetic treatment according to local practice.
8. Opioid-induced muscle rigidity is defined as increased muscle tone or breathing difficulties related to opioid infusion. Management includes reducing or interrupting the infusion of remifentanil; life-threatening rigidity will be treated immediately with airway support and administering a rapidly acting neuromuscular blocker, if required.
9. Neurological deterioration is defined as a GCS decrease of ≥ 2 points, the emergence of a new focal neurological deficit, seizure, suspected intracranial hypertension, or another concerning neurological change. Management includes immediate ICU and neurosurgical evaluation, interruption or adjustment of the protocol if needed, and urgent CT or MRI when clinically indicated.
10. Accidental extubation is defined as an unplanned removal of an endotracheal or tracheostomy tube. Accidental catheter or drain removal is defined as unplanned removal of a vascular catheter, gastric tube, urinary catheter, surgical drain, ventricular drain, or other clinically relevant device. Each event, the need for replacement, and the timing will be recorded separately.

The principal investigator and clinical team will review safety events regularly. Serious adverse events, protocol interruptions for safety, and unexpected drug-related adverse reactions will be reported to the IRB within 24 h. Since this is a small single-arm exploratory study using approved ICU medications, an independent data monitoring committee is not planned. However, protocol-level safety review will be triggered if any of the following occurs: two or more severe respiratory depression events that are possibly related to remifentanil; two or more severe opioid-related hypotension or bradycardia events requiring vasopressor escalation or atropine administration; any unexpected serious adverse drug reaction; two or more urgent rescue sedation events for uncontrolled agitation within a rolling 10-patient period; or any neurological deterioration considered possibly related to the protocol. A triggered review may lead to protocol suspension, dose-range modification, additional monitoring requirements, or IRB consultation.

### 2.9 Data management and confidentiality

Each participant will be assigned a coded study identification number. Research data will be entered into secure electronic case report forms with restricted access, individual usernames, passwords, and audit trails. Identifiable information will be stored separately from research data. Data quality will be maintained through range checks, logic checks, source-data verification, review of missing or inconsistent values, and regular monitoring by trained study staff and the IRB-designated monitor. Protocol deviations will be recorded and classified as major or minor. Before analysis, the database will be cleaned and locked. De-identified data may be made available from the corresponding author on reasonable request after study completion with appropriate institutional approval.

### 2.10 Sample size

In this study, Power Analysis and Sample Size (PASS) version 15.0 software was used for sample-size calculation. Since robust historical data are not available for an analgesia-first strategy without routine hypnotic sedatives in post-craniotomy ICU patients, this study is framed as an exploratory feasibility and safety study rather than a single-arm non-inferiority trial. The planned sample size of 65 participants is based on feasibility at the study center and the need to estimate the primary success proportion with acceptable precision for planning a future controlled study. For example, if the observed success proportion is approximately 80%, a sample of 65 would provide a two-sided 95% confidence interval with an approximate half-width of about 10 percentage points. This sample is also expected to provide preliminary safety data in a selected post-craniotomy population.

### 2.11 Statistical analysis

The full-analysis set will include all eligible enrolled patients who start remifentanil under the study protocol. The per-protocol set will exclude patients with major protocol violations. The primary endpoint will be reported as a proportion with a two-sided 95% confidence interval calculated using the exact binomial or Wilson method. In the main analysis, rescue hypnotic sedation, protocol discontinuation for safety, withdrawal before primary endpoint ascertainment, death, and an unassessable primary outcome will be classified conservatively as non-success. Sensitivity analyses will evaluate the effects of early ICU discharge before 24 h and missing primary outcome data.

Continuous variables will be summarized as means with standard deviations or medians with interquartile ranges, as appropriate. Categorical variables will be summarized as counts and percentages. ICU cost, duration of intubation, ventilator-free days, ICU length of stay, hospital length of stay, and drug exposure will be analyzed descriptively as continuous or count outcomes. Kaplan–Meier methods will be used only for outcomes with a genuine time-to-event structure and a prespecified censoring rule. Exploratory subgroup descriptions may be performed according to ventilation status at enrollment, surgical site, and baseline RASS category, without formal inferential claims. All analyses will be performed using IBM SPSS Statistics version 24.0 or later and/or R statistical software.

### 2.12 Patient and public involvement

Patients and the public were not involved in the design, conduct, reporting, or dissemination plans of this protocol.

## 3 Discussion

This protocol addresses a practical and clinically important problem in postoperative neurocritical care: managing pain and agitation while preserving the ability to perform timely neurological assessments (9–13). The proposed intervention is deliberately described as an analgesia-first strategy without routine hypnotic sedatives, rather than indicating a true absence of sedation. This terminology is important because remifentanil may affect arousal and the target for the RASS includes light sedation (19, 20, 23). The clinical goal is not to keep every patient fully awake at all times, but to avoid unnecessary hypnotic sedation while maintaining comfort, safety, and assessability.

In recent years, analgesia and sedation have become an important component of overall treatment in ICU care (26). The available evidence from general ICU settings supports interest in minimizing sedation, but it does not demonstrate specific benefits for post-craniotomy patients. Many guidelines and studies have proposed an analgesic-first sedation strategy and have reiterated the importance of analgesic treatment in the ICU (2, 4). The Society of Critical Care Medicine (SCCM) recommends an analgesia-based sedation approach in the general critical care population (5). Studies suggest that the use of analgesia-based sedation can help achieve sedation goals, decrease the amount of sedatives used, shorten ICU length of stay, and reduce ventilator days. In 2013 (2), a guideline was introduced proposing an early goal-oriented sedation strategy (early goal-directed sedation [EGDS]), which is based on analgesia with light sedation as the target. In 2016 (4), the chairman of Vincent of the European Critical Care Medical Association first proposed the eCASH concept based on a cluster sedation strategy. Its core contents include early implementation, prioritizing comfort and analgesia, minimizing sedation, and maximizing humanistic care. The 2010 single-center no-sedation trial not only suggested more ventilator-free days and shorter ICU and hospital stays but also resulted in a higher occurrence of agitated delirium (14). The larger NONSEDA trial did not show a significant difference in 90-day mortality or ventilator-free days between non-sedation and light sedation, and a substantial proportion of patients assigned to non-sedation ultimately required sedatives (15). These findings support the need for careful rescue criteria rather than rigid avoidance of sedatives. As neurocritical care patients were largely excluded from these studies, the feasibility and safety of sedation-minimization strategies in this population remain uncertain.

Neurocritical care introduces additional complexity. Sedation may be needed for intracranial pressure control, seizure management, oxygenation, mechanical ventilation, and prevention of secondary brain injury (10–13). Conversely, unnecessary sedation can obscure neurological examinations and may contribute to delirium, prolonged ventilation, immobility, and delayed recovery (9–12). Therefore, this study excludes severe traumatic brain injury, intracranial hypertension, status epilepticus, severe hypoxemia, therapeutic hypothermia, or other indications for deep sedation. The protocol also includes explicit neurological safety endpoints and criteria for urgent neuroimaging, rescue sedation, protocol discontinuation and safety review.

In the study, patients who had a RASS score > + 1 and an anticipated ICU stay of more than 24 h after neurosurgical craniotomy are included. These neurosurgical patients are more vulnerable to stress caused by agitation due to longer anesthesia duration, delayed extubation, pain, and post-craniotomy frontal pneumocephalus (6, 8). Patients who need deep sedation, particularly those with severe traumatic brain injury or intracranial hypertension, are excluded.

The strategy that focuses on using a short-acting opioid infusion (remifentanil or fentanyl) alone to manage pain and discomfort may result in a reduction in the duration of mechanical ventilation and ICU length of stay. Remifentanil was selected because its rapid onset and offset allow frequent titration and reassessment (18–21). In post-craniotomy ICU care, this pharmacological profile is attractive because neurological signs may need to be evaluated repeatedly (27–29) and promptly. Nevertheless, opioid-related adverse effects such as respiratory depression, hypotension, bradycardia, nausea, vomiting, and muscle rigidity require close monitoring (19–21). The protocol therefore defines operational thresholds for dose reduction (27–29), interruption, discontinuation, and safety review.

This study has limitations. It is a single-center, open-label, and single-arm trial; therefore, it cannot establish comparative effectiveness against standard analgesia and sedation care. The sample size was designed for feasibility and preliminary safety estimation, rather than for definitive efficacy. The enrolled population was selectively chosen and excludes patients who require deep sedation. As a result, the findings may not be applicable to patients with severe brain injury, uncontrolled intracranial hypertension, status epilepticus, or severe respiratory failure. However, the study may provide important preliminary data for designing a future multicenter controlled trial and for refining patient selection, rescue criteria, and neurological safety monitoring.
