# Supplementary material for: Feasibility and safety of an analgesia-first strategy without hypnotic sedatives in adult patients admitted to the intensive care unit after neurosurgical craniotomy: a protocol for a single-arm, single-center exploratory prospective study
Source: Front Med (Lausanne). 2026 Jun 10;13:1848074. doi: 10.3389/fmed.2026.1848074 (PMC13291133; doi:10.3389/fmed.2026.1848074)
Supplement: Supplementary file 1 [file Presentation_1.pdf]

SPIRIT

STANDARD PROTOCOL ITEMS: RECOMMENDATIONS FOR INTERVENTIONAL TRIALS

SPIRIT 2013 Checklist: Recommended items to address in a clinical trial protocol and related documents*

Section/item	ItemNo	Checklist item and manuscript information	Page
**Administrative information**			
Title	1	Descriptive title identifying the study design, population, interventions, and, if applicable, trial acronym	2.1 Study design and trial status
Trial registration	2a	Registered at ClinicalTrials.gov: NCT06727435. Registry status and dates are described in the manuscript.	2.1 Study design and trial status
	2b	WHO Trial Registration Data Set items are available in the ClinicalTrials.gov record; key registry details, status, estimated enrolment, and dates are summarized.	2.1 Study design and trial status
Protocol version	3	2026-5-17. Version 3.0	2 Materials and methods
Funding	4	No financial support was received for the research, authorship, or publication of the article.	Funding
Roles and responsibilities	5a	Protocol contributors, affiliations, and author contributions are provided, including conceptualization, methodology, investigation, data curation, supervision, project administration, and writing roles.	Author contributions
	5b	Investigator-initiated study. Corresponding author/contact: Guang-Zhi Shi, Beijing Tiantan Hospital, Capital Medical University; e-mail: shiguangzhi@bjtth.org.	Title page
	5c	No external sponsor or funder. The funding statement declares no financial support; therefore no funder role in study design, data handling, analysis, reporting, or publication decision applies.	Funding

	5d	The principal investigator and clinical team oversee safety review; an independent DMC is not planned because this is a small single-arm exploratory study using approved ICU medications.	Funding
**Introduction**			
Background and rationale	6a	Rationale is provided for analgesia-first care, sedation minimization, neurocritical-care-specific safety concerns, and remifentanil-based titration in post-craniotomy ICU patients.	1 Introduction
	6b	N/A - no comparator group is used. The study is a single-arm exploratory feasibility and safety protocol intended to generate preliminary data for a future controlled study.	2.5Intervention
Objectives	7	The objective is to explore the feasibility and safety of an analgesia-first strategy without routine hypnotic sedatives in adult ICU patients after neurosurgical craniotomy.	1 Introduction
Trial design	8	Single-centre, single-arm, open-label, exploratory prospective clinical study.	2.1 Study design and trial status
**Methods: Participants, interventions, and outcomes**			
Study setting	9	This is a single-centre, single-arm, open-label, exploratory prospective clinical study conducted in the Department of Critical Care Medicine, Beijng Tiantan Hospital, Capital Medical University, China, a major neurosurgical centre.	2.1 Study design and trial status
Eligibility criteria	10	Inclusion and exclusion criteria are listed, including adult post-craniotomy ICU admission, anticipated ICU stay, RASS threshold, assessment feasibility, and exclusions for deep sedation needs and safety risks.	2.3 Inclusion criteria<br>2.4 Exclusion criteria

Interventions	11a	Analgesia-first strategy without routine hypnotic sedatives; non-pharmacological measures, remifentanil initiation/titration, morphine rescue, rescue hypnotic sedation criteria, and monitoring are described.	2.5Intervention Table2
	11b	Dose reduction, interruption, discontinuation, protocol failure, rescue sedation, and safety discontinuation criteria are described.	2.5Intervention
	11c	RASS/CPOT targets, scheduled assessments, respiratory/hemodynamic monitoring, protocol algorithm, case report forms, and protocol deviation recording are described.	2.5Intervention
	11d	Permitted concomitant care includes treatment of reversible causes, non-pharmacological measures, antipsychotics for delirium when clinically required, airway/ventilatory support, antiemetics, bowel regimen, and standard ICU care. Rescue hypnotic sedatives are permitted for safety and count as non-success	2.5Intervention
Outcomes	12	Primary feasibility endpoint, key safety endpoints, secondary endpoints, assessment windows, operational RASS rule, and endpoint hierarchy are specified.	2.6 Outcomes
Participant timeline	13	Schedule of enrolment, intervention, assessments, ICU discharge, and hospital discharge/death is provided; flowchart is included.	2 Materials and methods Table1 Figure1
Sample size	14	Planned sample size of 65 participants is based on feasibility and precision for estimating the primary success proportion; approximate 95% CI half-width is described	2.10 Sample size
Recruitment	15	All ICU admissions after neurosurgical craniotomy will be screened; feasibility is based on the study centre and target enrolment of 65 participants.	2.10 Sample size

Methods: Assignment of interventions (for controlled trials)

Allocation:

Sequence generation	16a	N/A - this is a single-arm study with no random allocation sequence.	2.1 Study design and trial status

Allocation concealment mechanism	16b	N/A - this is a single-arm study with no allocation concealment.	2.1 Study design and trial status
Implementation	16c	N/A - no allocation is performed. Screening and consent procedures for enrolled participants are described.	2.1 Study design and trial status
Blinding (masking)	17a	N/A - the study is open-label and single-arm; participants and clinicians are not blinded.	2.1 Study design and trial status
	17b	N/A - no blinding is used, so emergency unblinding procedures do not apply.	2.1 Study design and trial status

Methods: Data collection, management, and analysis

Data collection methods	18a	Baseline, peri-intervention, neurological, respiratory, hemodynamic, drug exposure, adverse event, CAM-ICU, ICU discharge, and hospital outcome data are prospectively collected by trained research staff using standardized case report forms.	2.7 Data collection and assessment schedule
	18b	Participants are followed until hospital discharge or death. Withdrawal rights, protocol deviation handling, early ICU discharge handling, and missing primary outcome sensitivity analyses are described.	2.7 Data collection and assessment schedule
Data management	19	Coded study IDs, secure electronic case report forms, restricted access, separate identifiable information, audit trails, range checks, logic checks, source-data verification, monitoring, database cleaning, and locking are described.	2.7 Data collection and assessment schedule
Statistical methods	20a	Primary endpoint will be reported as a proportion with two-sided 95% exact binomial or Wilson confidence interval; secondary outcomes will be analyzed descriptively as appropriate.	2.11 Statistical analysis
	20b	Sensitivity analyses for early ICU discharge and missing primary outcome data; exploratory subgroup descriptions by ventilation status, surgical site, and baseline RASS category.	2.11 Statistical analysis

	20c	Full-analysis set, per-protocol set, conservative classification of non-success, and sensitivity analyses for missing or early-discharge primary outcome data are defined.	2.11 Statistical analysis
**Methods: Monitoring**			
Data monitoring	21a	No independent DMC is planned; rationale and protocol-level safety review by the principal investigator and clinical team are described.	2.9 Data management and confidentiality
	21b	No formal efficacy interim analysis is planned. Protocol-level safety review triggers and potential actions, including suspension, dose-range modification, additional monitoring, or IRB consultation, are specified.	2.9 Data management and confidentiality
Harms	22	Plans for collecting, grading, reporting, and managing adverse events are described, including respiratory depression, hemodynamic events, delirium, vomiting/aspiration risk, opioid-induced rigidity, device removal, neurological deterioration, SAE reporting, and safety review triggers.	2.8 Adverse events, rescue management, and safety oversight
Auditing	23	Data quality and trial conduct will be monitored through source-data verification, review of missing or inconsistent values, regular monitoring by trained study staff, and an IRB-designated monitor.	2.9 Data management and confidentiality
**Ethics and dissemination**			
Research ethics approval	24	Approved by the Institutional Review Board of Beijing Tiantan Hospital, Capital Medical University (KY 2024-168-02); conducted according to the Declaration of Helsinki and institutional requirements.	Ethics statement
Protocol amendments	25	Recruitment is scheduled to start in August 2026 after the publication of this protocol. The registry entry will be updated before enrolment and publication to align with the revised title, exploratory study design, primary endpoint, recruitment status, and timeline. And the revised protocol will submit to IRB.	2.1 Study design and trial status

Consent or assent	26a	Written informed consent will be obtained preoperatively where feasible; legally authorized representatives may consent for patients lacking capacity; patients regaining capacity will be informed and asked to confirm participation.	2.2 Informed consent
	26b	N/A - no ancillary biological specimen or genetic/molecular study is planned in this protocol	NA<br>2.2 Informed consent
Confidentiality	27	Coded study IDs, restricted-access electronic case report forms, separate storage of identifiable information, usernames/passwords, audit trails, and de-identified data sharing safeguards are described	2.7 Data collection and assessment schedule
Declaration of interests	28	The authors declare no competing interests.	Conflict of interest
Access to data	29	De-identified data may be available from the corresponding author on reasonable request after study completion and institutional approval; no limiting contractual agreement is reported.	Publisher's note
Ancillary and post-trial care	30	Trial-related adverse events will be managed clinically according to ICU practice and reported to the IRB when serious. No separate post-trial intervention is planned because the intervention uses approved ICU medications during hospitalization.	Publisher's note
Dissemination policy	31a	Results will be disseminated through peer-reviewed publication and conference presentation.	Publisher's note
	31b	Authorship/contribution roles are provided; no professional medical writer is reported. Generative AI was used only for English language editing, with author responsibility retained.	Generative AI statement
	31c	A SPIRIT checklist will be submitted as supplementary material. De-identified data may be available from the corresponding author on reasonable request after study completion and institutional approval; public access to statistical code is not specified.	2.1 Study design and trial status

Appendices

Informed consent materials	32	Model consent form is not included in the manuscript file. Consent procedures for participants and legally authorized representatives are described.	2.2 Informed consent
Biological specimens	33	N/A - no biological specimens are collected, stored, or evaluated for genetic or molecular analysis in the current trial.	NA

*It is strongly recommended that this checklist be read in conjunction with the SPIRIT 2013 Explanation & Elaboration for important clarification on the items. Amendments to the protocol should be tracked and dated. The SPIRIT checklist is copyrighted by the SPIRIT Group under the Creative Commons "Attribution-NonCommercial-NoDerivs 3.0 Unported" license.
